# Free Radical Scavenging Activity of Drops and Spray Containing Propolis—An EPR Examination

**DOI:** 10.3390/molecules22010128

**Published:** 2017-01-13

**Authors:** Pawel Olczyk, Katarzyna Komosinska-Vassev, Pawel Ramos, Lukasz Mencner, Krystyna Olczyk, Barbara Pilawa

**Affiliations:** 1 Department of Community Pharmacy, School of Pharmacy and Division of Laboratory Medicine in Sosnowiec, Medical University of Silesia in Katowice, Kasztanowa 3, 41-200 Sosnowiec, Poland; 2 Department of Clinical Chemistry and Laboratory Diagnostics, School of Pharmacy and Division of Laboratory Medicine in Sosnowiec, Medical University of Silesia in Katowice, Jedności 8, 41-200 Sosnowiec, Poland; kvassev@sum.edu.pl (K.K.-V.); lukasz.mencner@gmail.com (L.M.); olczyk@sum.edu.pl (K.O.); 3 Department of Biophysics, School of Pharmacy and Division of Laboratory Medicine in Sosnowiec, Medical University of Silesia in Katowice, Jedności 8, 41-200 Sosnowiec, Poland; pawelramos@sum.edu.pl (P.R.); bpilawa@sum.edu.pl (B.P.)

**Keywords:** antioxidant, propolis, drops, spray, heating, UV-irradiation, EPR spectroscopy

## Abstract

The influence of heating at a temperature of 50 °C and UV-irradiation of propolis drops and spray on their free radical scavenging activity was determined. The kinetics of interactions of the propolis samples with DPPH free radicals was analyzed. Interactions of propolis drops and propolis spray with free radicals were examined by electron paramagnetic resonance spectroscopy. A spectrometer generating microwaves of 9.3 GHz frequency was used. The EPR spectra of the model DPPH free radicals were compared with the EPR spectra of DPPH in contact with the tested propolis samples. The antioxidative activity of propolis drops and propolis spray decreased after heating at the temperature of 50 °C. A UV-irradiated sample of propolis drops more weakly scavenged free radicals than an untreated sample. The antioxidative activity of propolis spray increased after UV-irradiation. The sample of propolis drops heated at the temperature of 50 °C quenched free radicals faster than the unheated sample. UV-irradiation weakly changed the kinetics of propolis drops or spray interactions with free radicals. EPR analysis indicated that propolis drops and spray should not be stored at a temperature of 50 °C. Propolis drops should not be exposed to UV-irradiation.

## 1. Introduction

Propolis (bee glue) is a well-known, complex, resinous, natural material collected by honeybees (*Apis mellifera L.*) from various plant sources such as leaves, buds, barks and exudates [1,2,3]. This natural mixture exhibits a wide variety of botanical sources and chemical compositions, depending on the bee species, location of the production and the collection season [4,5,6]. The predominant sources of Polish propolis, as in other countries from the temperate zones, are *Poplar* sp.—*Populus alba*, *Populus tremula*, *Populus nigra*—secondarily bee glue is collected by bees from *Betula pendula*, *Alnus glutinosa*, *Pinus sylvestris* and *Salix* sp. *L. buds* [7,8,9]. Poplar propolis, used in the present study, contains 50% of resins and vegetable balms, 30% of wax, 10% of aromatic and essential oils, 5% of pollen, and 5% of additional active substances [10]. In addition to the abovementioned main constituents of propolis—resins, wax, essential oils and pollen—so far 300 active compounds with proven biological activity have been isolated. The most important of them are the following compounds: phenolic acids (benzoic, cinnamic, caffeic and ferulic acids), phenols (vanillin and eugenol) coumarin (esculetin), flavones (chrysin, tectochrysin, luteolin, apigenin, acacetin), flavonols (butelenol, galangin, kaempherol, quercetin, rhamnocitrin, izalpinin), and flavanones (pinostrobin, pinocembrin). There are also terpene compound (bisabolol), fatty acids and their esters (linolenic, oleic, butyric and valeric acids), alcohols (cetyl, myricyl, mannitol, inositol), micronutrients (calcium, magnesium, manganese, zinc, copper, iron, silicon, aluminum, cobalt, zirconium, ruby, selenium), vitamins (B_1_, B_2_, B_6_, C, E), amino acids (aspartic acid, glutamic acid, tryptophan, phenylalanine, leucine, cysteine, proline) and enzymes (succinate dehydrogenase, glucose-6-phosphatase, adenosine triphosphatase, acid phosphatase) [11,12,13].

Different combinations of the active compounds listed above that mediate the complex and interrelated mechanisms of action of propolis are essential for the antibacterial, antiviral, antifungal, as well as free radical scavenging action [3,9,11,13,14]. Nowadays however, particular attention is paid to its antimicrobial and antioxidative activity [1,8,13]. The free radical scavenging capacity of propolis formulations seems to be associated with the presence of phenolic compounds, especially flavonoids such as apigenin, tectochrysin, chrysin, galangin, pinocembrin, genkwanin, kaempherol, and 5-hydroxy-4′,7-dimethoxyflavone, pilloin and pinostrobin chalcone. Different mechanisms could be responsible for the antioxidative properties of the phenolic compound [3,6,9]. These natural agents can inhibit the free radicals generation, interrupt the free radical chain reactions, chelate transition metal ions taking part in the oxidative processes as well as inhibit the activity of xanthine oxidase and NADPH oxidase, the enzymes responsible for the appearance of superoxide anion radical [3,6,9].

Propolis formulations, due to their safety of use and numerous health-promoting properties, are being more and more commonly used in prevention and treatment of many diseases [1,8,13], including mucous and dermal inflammatory pathologies. Less attention has been paid to the anti-inflammatory or anesthetic properties of propolis formulations available on the market, i.e., sprays and drops [2,15]. The mentioned preparations are predominantly prepared using ethanol as solvent, which, unlike propolis water formulations described in this study, is unfortunately responsible for irritating and drying effects on mucosal biological membranes, being also unsuitable for use in pediatrics, ophthalmology, and in case of alcohol intolerance [2,16].

In this work the hypothesis about the antioxidative character of propolis drops and spray was checked. Products of different concentrations of propolis, i.e., 10% for drops and 20% for spray, were chosen in order to evaluate if the overall antioxidant activity depends on the propolis concentration in the sample. The aim of this study was also to determine the influence of higher temperature and UV-irradiation on the free radical scavenging activity of propolis drops and propolis spray. Kinetics of interactions with free radicals of the untreated propolis samples and the samples affected by the physical factors (thermal factor and ultraviolet irradiation) were analyzed. The free radical scavenging activity of propolis drops and spray has not been examined by the EPR method so far. This knowledge about quenching of free radicals by propolis drugs is of practical importance in choosing storage conditions.

## 2. Results and Discussion

Electron paramagnetic resonance measurements of the DPPH spectra pointed out that all the examined samples containing propolis, in both drop and spray form, interacted with DPPH free radicals and revealed their scavenging activity. The phenolic-type active compounds seem to be a major determinant of the antioxidative potential in bee-glue preparations. Among the ones identified in Polish propolis the following flavonoids can be mentioned: apigenin tectochrysin, chrysin, galangin, pinocembrin, genkwanin, kaempherol and 5-hydroxy-4′,7-dimethoksyflavonepilloin and pinostrobin chalcone [9]. The mentioned phenolic active molecules are major determinants of the antioxidative potential in the used propolis preparations, being responsible for the scavenging of DPPH radicals. Free radical scavenging activity had an effect on the decrease of the DPPH EPR lines. The decrease of the DPPH EPR lines increased with increasing duration of the interaction with the propolis samples, and after a characteristic time for individual samples it became constant. These constant values corresponded to the stabilization of DPPH free radicals’ interactions with propolis drops or spray. The g-value for DPPH EPR lines was 2.0036.

**Figure 1:**
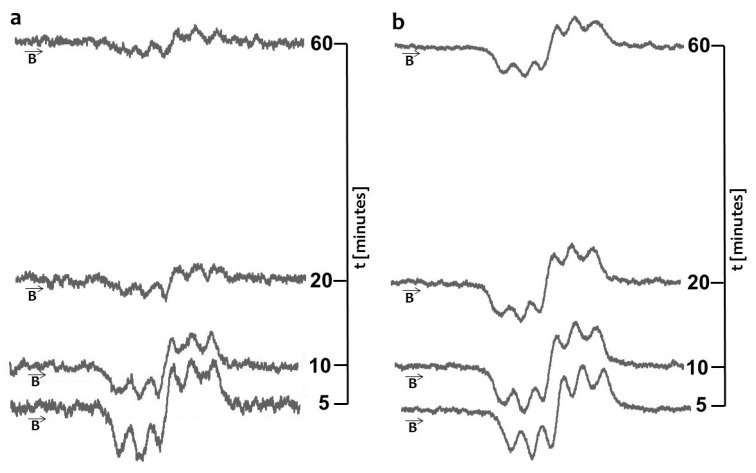
EPR spectra of DPPH interacting with unheated and non-irradiated (**a**) propolis drops; and (**b**) propolis spray for 5 min, 10 min, 20 min, 60 min. **B**—magnetic induction.

The quenching of the EPR spectra of DPPH in contact with the original, unheated and non-irradiated propolis drops with increasing interaction time is shown in Figure 1a. The scavenging effect on DPPH free radicals and propolis spray is visible in Figure 1b. The model DPPH free radicals were scavenged by both original propolis samples—drops and spray. Free radicals are scavenged by antioxidative substances [17,18,19], thus the antioxidative character of the tested propolis samples was confirmed.

**Figure 2:**
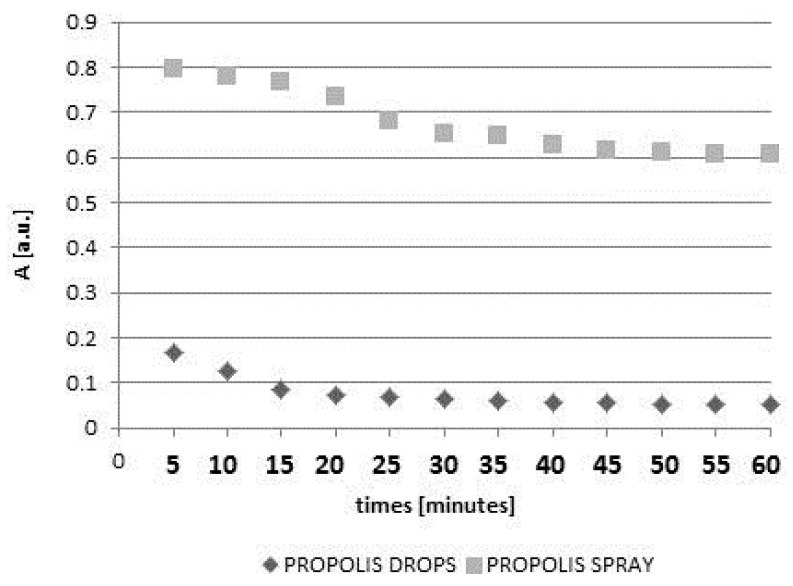
The influence of time (t) of interactions on amplitudes (A) of EPR lines of DPPH in contact with unheated and non-irradiated propolis drops and spray.

The kinetics of DPPH free radicals interactions with unheated and non-irradiated drops and spray containing propolis are shown in Figure 2.

**Figure 3:**
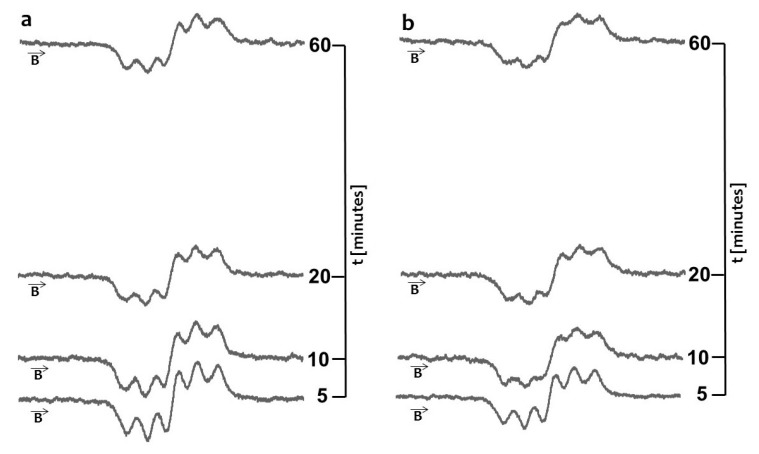
EPR spectra of DPPH interacting with (**a**) propolis drops and (**b**) propolis spray heated at the temperature 50 °C for 5 min, 10 min, 20 min, 60 min. **B**—magnetic induction. Time of heating was 30 min.

**Figure 4:**
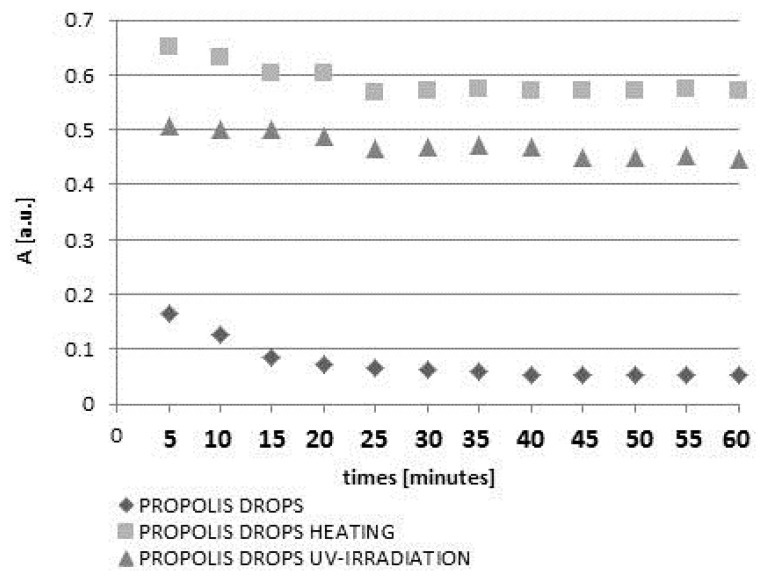
The effect of both heating at 50 °C and UV-irradiation on changes of amplitudes (A) of EPR lines of DPPH in contact with propolis drops with increasing interaction time (t). Times of both heating and UV-irradiation were 30 min.

**Figure 5:**
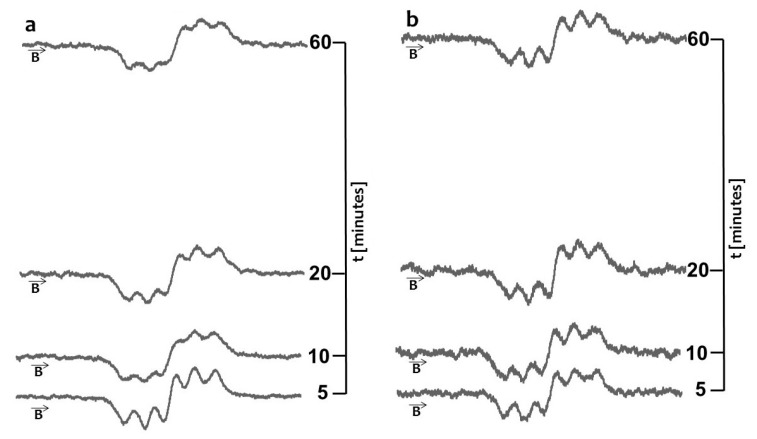
EPR spectra of DPPH interacting with (**a**) propolis drops and (**b**) propolis spray UV-irradiated for 5 min, 10 min, 20 min, 60 min. **B**—magnetic induction. Time of UV-irradiation was 30 min.

In the first stage the amplitudes (A) of the DPPH lines decreased with increasing duration of the contact of the model free radical with propolis drops or propolis spray. However, after the amplitudes (A) reached the constant value, they did not change during 60 min of observations. The moment of stabilization appeared after 40 min for drops and after 45 min for spray. The scavenging activity of 10% propolis drops was much stronger than the activity of 20% propolis spray. The obtained results indicate that the overall antioxidant activity of water propolis formulation is not directly proportional to the propolis concentration in the sample. It is widely accepted that the propolis antioxidant activity depends on many factors including environmental conditions such as botanical and geographical origin, the types of plant species, age and status of the plant as well as the chemical composition and the extraction method [5,8,9]. Taking this into account as well as the problems with standardization of propolis formulations, EPR spectroscopy, which gives direct information about the overall antioxidative activity in the sample, could be proposed as a method of choice for the evaluation and comparison of antioxidant properties of various propolis formulations. Both heating at a temperature of 50 °C and UV-irradiation changed the free radical scavenging activity of the drops and spray containing propolis. The quenching of the EPR spectra of DPPH free radicals with propolis drops heated at the temperature of 50 °C and UV-irradiated drops containing propolis, for 5, 10, 20, and 60 min of interaction is presented in Figure 3a and Figure 5a, respectively. The kinetics of the interactions of DPPH with the original untreated propolis drops, and the heated or UV-irradiated propolis drops, is compared in Figure 4. The scavenging activity of propolis drops against free radicals decreased after heating at the temperature of 50 °C and after exposure to UV (Figure 4). The time until stabilization of the amplitudes (A) values was different for the original, heated, or UV-irradiated propolis drops (Figure 4).

**Figure 6:**
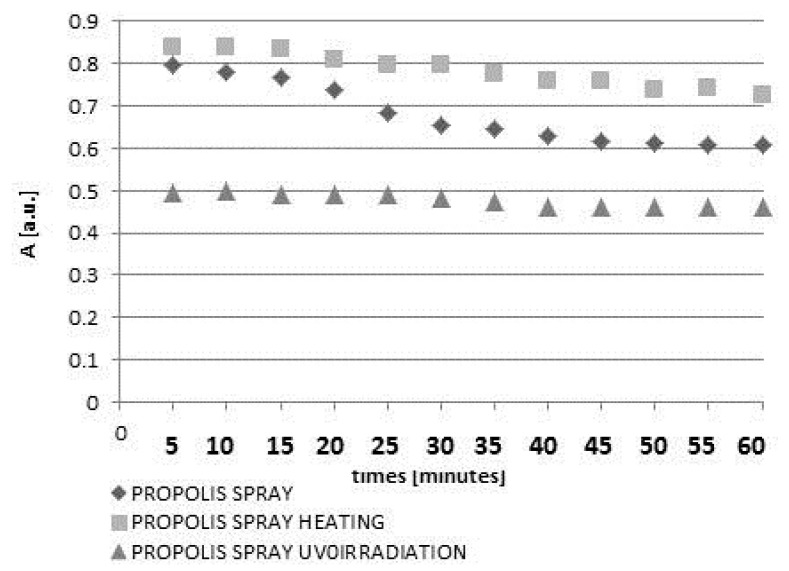
The effect of heating at the temperature of 50 °C and UV-irradiation on changes of amplitudes (A) of EPR lines of DPPH in contact with propolis spray with increasing interaction time (t). Times of both heating and UV-irradiation were 30 min.

The scavenging activity of propolis spray against free radicals depended on the storage conditions of the samples. The quenching of the EPR spectra of DPPH free radicals by propolis spray heated at the temperature of 50 °C and by the UV-irradiated samples, for 5, 10, 20, and 60 min of interactions are presented in Figure 3b and Figure 5b, respectively. The comparison of the kinetics of interactions of DPPH with propolis spray for the original untreated propolis spray, and the heated and UV-irradiated spray samples, was done in Figure 6. The heating of propolis spray at the temperature of 50 °C decreased its interactions with DPPH free radicals (Figure 6). The free radical scavenging activity of propolis spray after UV-irradiation was higher than in the case of the original untreated sample (Figure 6).

**Figure 7:**
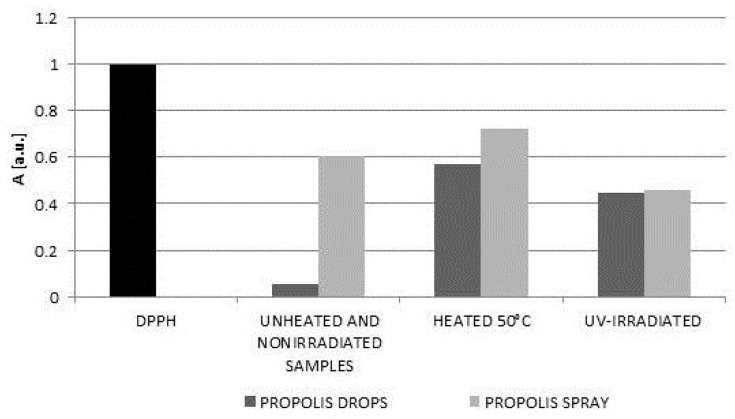
Comparison of the amplitudes (A_min_) (±0.01 a.u.) of EPR lines of DPPH in contact with drops and spray containing propolis for: the unheated and non-irradiated samples, the heated samples, and the UV-irradiated samples. The data for interaction time of DPPH with the propolis samples equal 60 min.

The interactions of DPPH free radicals changed by heating or exposure to UV on storage of propolis samples. High temperatures may be responsible for the destruction of propolis active ingredients responsible for the antioxidative potential, i.e., the phenolic compounds, ascorbic acid and cysteine (a sulfur-containing amino acid) and tryptophan [20,21,22]. In turn, UV radiation may be responsible for the degradation of phenols in aqueous solutions [23]. The values of the minimal amplitudes (A_min_) of DPPH free radicals in contact with the original untreated propolis drops and spray, the propolis samples heated at the temperature of 50 °C, and the UV-irradiated propolis samples, are compared in the diagram shown in Figure 7. The free radical scavenging activity was higher for the lower values of the minimal amplitudes (A_min_). The minimal values of the amplitudes (A_min_) for DPPH in contact with propolis drops decreased in the following order: propolis drops heated at the temperature of 50 °C > UV-irradiated propolis drops > unheated and non-irradiated propolis drops (Figure 7). The values of (A_min_) for DPPH in contact with propolis spray decreased in the order: propolis spray heated at the temperature of 50 °C > unheated and non-irradiated propolis spray > UV-irradiated propolis spray (Figure 7). Heating of both propolis drops and spray decreased their scavenging activity against free radicals. UV-irradiation decreased and increased free radical scavenging activity of propolis drops and propolis spray, respectively.

**Figure 8:**
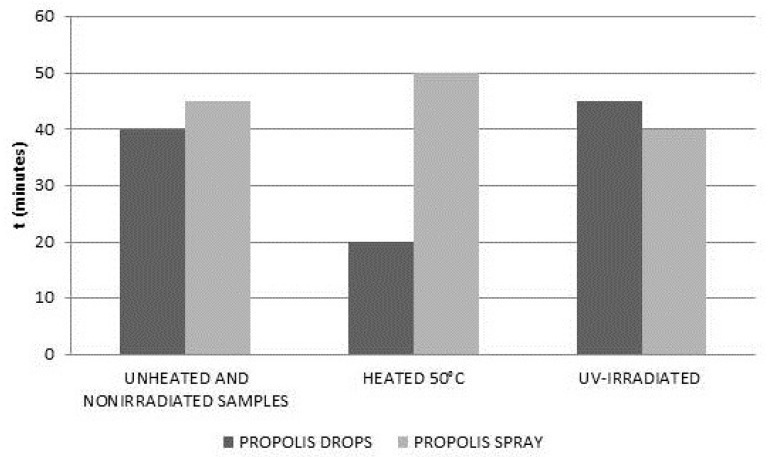
Comparison of the stabilization times (t) (±5 min) of DPPH interactions with drops and spray containing propolis for: the unheated and non-irradiated samples, the heated samples, and the UV-irradiated samples.

The times of stabilization of the DPPH free radicals interactions with propolis drops and propolis spray, were determined from the kinetics of these interactions (Figure 4 and Figure 6) were compared in Figure 8. In Figure 8 the stabilization times for the original samples, the samples heated at the temperature of 50 °C, and UV-irradiated samples were taken into account. 

Only slight changes in the stabilization times were observed after UV irradiation of propolis drops and propolis spray. A significantly faster stabilization of the interactions of propolis drops with DPPH free radicals was observed after heating these samples at the temperature of 50 °C. After heating the propolis drops, the stabilization time decreased from 40 min (for the original untreated sample) to 20 min. Interactions of propolis drops with free radicals dropped considerably faster after heating. After heating the propolis spray, the stabilization time increased from 45 min (for the original untreated sample) to 50 min. Interactions of propolis spray with free radicals declined slower after heating.

Electron paramagnetic resonance spectroscopy and the model DPPH free radicals were used in the examination of the interactions of propolis drops and propolis spray with free radicals. The antioxidative character of the tested propolis samples was brought to light. The effects of storage conditions at higher temperature and UV-irradiation on free radical scavenging activity of propolis drops and propolis spray, were discussed. The results may be used in practice to optimize the storage conditions of propolis drops and propolis spray. It should also be noted that propolis antioxidative activity is strengthened in the case of water propolis formulations—in comparison to the alcoholic preparations—being mediated by the higher amount of phenolic compounds in the first mentioned market products [24]. Therefore, it can be pointed out that the bee glue antioxidative property is mediated by the reduction of the activity of several enzymes, which in turn prevents the production of reactive oxygen species. It occurs by scavenging, interrupting the phenomena that lead to the lipid peroxidation, and by chelating metal ions, mainly iron and copper, that are involved in the process of free radical creation or by stronger intensification of the action of other antioxidants in the case of propolis water extracts [24,25].

## 3. Materials and Methods

### 3.1. Propolis Samples

Two Polish propolis formulations, namely 10% drops (Apiterapia, norm no. 2009/01) and 20% propolis spray (Virdepol, norm no. RK/279829/2010)—the most commonly used propolis water extracts commercially available on the market—were examined in this study.

Different storage conditions of the propolis samples were considered. Samples stored at room temperature, at 50 °C, and exposed to UV were tested. The propolis drops and the liquid obtained from the propolis spray, were heated at the temperature of 50 °C for 30 min, because the temperature during the exposure of samples under sun irradiation may increase up to 50 °C. The free radical changes found after 30 min of heating indicate that we can expect the changes in the sample also after a longer time of heating. A professional hot air oven with air circulation produced by the Memmert Company (Schwabach, Germany) was used.

The propolis samples were exposed to UVA radiation with the wavelengths (λ) in the range 315–400 nm for 30 min. The irradiation was performed by a Medisun 250 (Schulze & Böhm, Brühl, Germany) system with four irradiators with a power of 20 W. The distance between the lamp and the propolis samples was 30 cm.

### 3.2. The Model Free Radicals

The free radical scavenging activity of propolis samples was examined by the use of paramagnetic 1,1-diphenyl-2-picrylhydrazyl (DPPH) molecules as the model free radicals. The unpaired electron in the DPPH molecule is located on the nitrogen (N) atom [26,27].

### 3.3. EPR Measurements

EPR spectra were measured for DPPH free radicals. DPPH and DPPH with the tested propolis samples were prepared in ethyl alcohol solution (95%). The solutions were placed in thin-walled glass tubes with the external diameter of 1 mm. EPR signals were not observed for the empty glass tubes, which indicated their high paramagnetic purity. The masses of DPPH were obtained by a Sartorius CPA (Goettingen, Germany).

An X-band (9.3 GHz) electron paramagnetic resonance spectrometer from the Radiopan firm (Poznań, Poland) was used in this study. Microwave frequency was measured by an MCM101 recorder produced by the EPRAD Company (Poznań, Poland). Magnetic modulation was 100 kHz and the total microwave power produced by the klystron in the microwave bridge was 70 mW. The applied attenuation of 15 dB changed the microwave power to 2.2 mW. This low value of the microwave power was used to avoid microwave saturation of the first-derivative EPR lines. EPR spectra were collected in the numerical form by the Rapid Scan Unit of the Jagmar Company (Kraków, Poland) used as the numerical data acquisition system.

The EPR spectra amplitudes (A) (±0.01 a.u.), and g-factors (±0.0002), were determined. The errors of the analyzed values were determined by the exact differential methods. The decrease of the amplitudes (A) of the DPPH EPR lines after addition of the propolis sample to the alcohol ethyl solution reflected the antioxidative character of the sample and its free radical scavenging activity [26,27,28,29,30,31]. The kinetics as well as the changes of amplitudes (A) of DPPH interacting with the individual propolis sample with increasing time up to 60 min by 5 min were determined. The g-factors were calculated from the resonance condition as [7,8,9,10]: g = hν/μ_B_B_r_, where: h—Planck constant, ν—microwave frequency, μ_B_—Bohr magneton, B_r_—induction of resonance magnetic field. The EPR measurements and analysis were performed using the professional spectroscopic programs of the Jagmar Company, LabView (National Instruments, Austin, TX, USA), and Origin (OriginLab, Wheeling, IL, USA).

## 4. Conclusions

Electron paramagnetic resonance spectroscopic studies of the EPR spectra of model DPPH free radicals illustrated the antioxidative character of both propolis drops and propolis spray. The propolis samples interacted with DPPH free radicals and they quenched DPPH EPR lines as the result of the free radical scavenging activity. A considerably higher free radical scavenging activity was obtained for the original, unheated and non-irradiated, propolis drops than for the original propolis spray. The free radical scavenging activity changed after heating at the temperature of 50 °C and after UV-irradiation of the tested samples. The free radical scavenging activity of propolis drops and propolis spray decreased after heating at the temperature of 50 °C, thus they should be protected from high temperature. UV-irradiation of propolis drops decreased their scavenging activity against free radicals, which indicated that they should not be stored with exposure to UV. An increase in free radical scavenging activity after UV-irradiation of propolis spray was observed. Heating propolis drops at a temperature of 50 °C strongly accelerated their interactions with free radicals. UV-irradiation did not considerably change the kinetics of propolis drops and propolis spray interactions with free radicals.

## References

[1] Sforcin J.M. (2016). Biological properties and therapeutic applications of propolis. Phytother. Res..

[2] Sosa S., Bornancin A., Tubaro A., Loggia R.D. (2007). Topical antiinflammatory activity of an innovative aqueous formulation of actichelated propolis vs. two commercial propolis formulations. Phytother. Res..

[3] Ristivojević P., Trifković J., Andrić F., Milojković-Opsenica D. (2015). Poplar-type propolis: Chemical composition, botanical origin and biological activity. Nat. Prod. Commun..

[4] Papachroni D., Graikou K., Kosalec I., Damianakos H., Ingram V., Chinou I. (2015). Phytochemical analysis and biological evaluation of selected African propolis samples from Cameroon and Congo. Nat. Prod. Commun..

[5] Huang S., Zhang C.P., Wang K., Li G.Q., Hu F.L. (2014). Recent advances in the chemical composition of propolis. Molecules.

[6] Machado B.A., Silva R.P., Barreto Gde A., Costa S.S., Silva D.F., Brandão H.N., Rocha J.L., Dellagostin O.A., Henriques J.A., Umsza-Guez M.A. (2016). Chemical composition and biological activity of extracts obtained by supercritical extraction and ethanolic extraction of brown, green and red propolis derived from different geographic regions in Brazil. PLoS ONE.

[7] Ristivojević P., Trifković J., Gašić U., Andrić F., Nedić N., Tešić Ž., Milojković-Opsenica D. (2015). Ultrahigh-performance liquid chromatography and mass spectrometry (UHPLC–LTQ/Orbitrap/MS/MS) study of phenolic profile of serbian poplar type propolis. Phytochem. Anal..

[8] Bankova V., Popova M., Trusheva B. (2014). Propolis volatile compounds: Chemical diversity and biological activity: A review. Chem. Cent. J..

[9] Kurek-Górecka A., Rzepecka-Stojko A., Górecki M., Stojko J., Sosada M., Świerczek-Zięba G. (2014). Structure and antioxidant activity of polyphenols derived from propolis. Molecules.

[10] Ristivojević P., Dimkić I., Trifković J., Berić T., Vovk I., Milojković-Opsenica D., Stanković S. (2016). Antimicrobial activity of Serbian propolis evaluated by means of MIC, HPTLC, bioautography and chemometrics. PLoS ONE.

[11] Olczyk M., Krysik K., Jędrusik P. (2014). Oparzenia—Charakterystyka i klasyfikacja. Czas. Aptek..

[12] Kędzia B., Kędzia A., Dudko P., Hołderna-Kędzia E. (2009). The activity of polish propolis on the pathogenic microorganisms of human and animal origin. Post. Fitoter..

[13] Miguel M.G. (2013). Chemical and biological properties of propolis from the western countries of the Mediterranean basin and Portugal. Int. J. Pharm. Pharm. Sci..

[14] Sforcin J.M., Bankova V. (2011). Propolis: Is there a potential for the development of new drugs?. J. Ethnopharmacol..

[15] Bogdanov S. (2016). Pollen: Production, Nutrition and Health: A Review. http://www.bee-hexagon.net/.

[16] Najafi M.F., Vahedy F., Seyyedin M., Jomehzadeh H.R., Bozary K. (2007). Effect of the water extracts of propolis on stimulation and inhibition of different cells. Cytotechnology.

[17] Kumazawa S., Ahn M.R., Fujimoto T., Kato M. (2010). Radical-scavenging activity and phenolic constituents of propolis from different regions of Argentina. Nat. Prod. Res..

[18] Aksoy L., Kolay E., Ağılönü Y., Aslan Z., Kargıoğlu M. (2013). Free radical scavenging activity, total phenolic content, total antioxidant status, and total oxidant status of endemic *Thermopsis turcica*. Saudi J. Biol. Sci..

[19] Peluso I., Miglio C., Morabito G., Ioannone F., Serafini M. (2015). Flavonoids and immune function in human: A systematic review. Crit. Rev. Food Sci. Nutr..

[20] Wojdyło A., Figiel A., Oszmiański J. (2007). Influence of temperature and time of apple drying on phenolic compounds content and their antioxidant activity. Pol. J. Food Nutr. Sci..

[21] Shi J. (2006). Functional Food Ingredients and Nutraceuticals: Processing Technologies.

[22] Wu G. (2010). Amino Acids: Biochemistry and Nutrition.

[23] Esplugas S., Chamarro E., Mokrini A. Degradation of Phenol in Aqueous Solutions Using Fe+3 and UV Radiation, Tecnologia em Tratamento de Água. http://www.snatural.com.br/PDF_arquivos/Efluente-Phenol-Degradation-UV-Fenton.pdf.

[24] Adomavičiūtė E., Stanys S., Žilius M., Briedis V. (2015). Formation and analysis of electrospun nonwoven mats from bicomponent PVA/Aqueous propolis nano-microfibres. Fibres Text. East. Eur..

[25] Kubiliene L., Laugaliene V., Pavilonis A., Maruska A., Majiene D., Barcauskaite K., Kubilius R., Kasparaviciene G., Savickas A. (2015). Alternative preparation of propolis extracts: Comparison of their composition and biological activities. BMC Complement. Altern. Med..

[26] Tirzitis G., Bartosz G. (2010). Determination of antiradical and antioxidant activity: Basic principles and new insights. Biochim. Pol. Acta.

[27] Bartosz G. (2006). Druga Twarz Tlenu: Wolne Rodniki w Przyrodzie.

[28] Molyneux P. (2004). The use of stable free radical diphenylpicrylhydrazyl (DPPH) for estimating antioxidant activity. Songklanakarin J. Sci. Technol..

[29] Eaton G.R., Eaton S.S., Salikhov K.M. (1998). Foundations of Modern EPR.

[30] Padmanabhan P., Jangle S.N. (2012). Evaluation of DPPH radical scavenging activity and reducing power of four selected medicinal plants and their combinations. Int. J. Pharm. Sci. Drug Res..

[31] Kurzeja E., Stec M., Ramos P., Pilawa B., Pawłowska-Góral K. (2013). Antioxidant properties of water extracts of sterilized and unsterilized Morus Alba L. Leaves. Int. J. Food Prop..

